# Health professional students at the University of Illinois Chicago (HOLISTIC) Cohort Study: A protocol

**DOI:** 10.1371/journal.pone.0269964

**Published:** 2022-08-30

**Authors:** Sunil R. Dommaraju, Stephanie Gordon Rivera, Ethan G. Rocha, Scott Bicknell, Daniel Loizzo, Ayesha Mohammad, Priya Rajan, Alexandria Seballos, Avisek Datta, Rashid Ahmed, Jerry A. Krishnan, Mary T. Keehn

**Affiliations:** 1 College of Medicine, University of Illinois Chicago, Chicago, Illinois, United States of America; 2 School of Public Health, University of Illinois Chicago, Chicago, Illinois, United States of America; 3 College of Applied Health Sciences, University of Illinois Chicago, Chicago, Illinois, United States of America; 4 College of Dentistry, University of Illinois Chicago, Chicago, Illinois, United States of America; 5 College of Nursing, University of Illinois Chicago, Chicago, Illinois, United States of America; 6 Population Health Sciences Program, Office of the Vice Chancellor for Health Affairs, University of Illinois Chicago, Chicago, Illinois, United States of America; 7 Interprofessional Practice and Education, Office of the Vice Chancellor for Health Affairs, University of Illinois Chicago, Chicago, Illinois, United States of America; Satyawati College (Eve.), University of Delhi, INDIA

## Abstract

**Objectives:**

The objectives of the HOLISTIC Cohort Study are to establish a prospective cohort study covering a period of three years that characterizes the health of students within and across health professional education programs at the University of Illinois Chicago (UIC) during the coronavirus disease 2019 (COVID-19) pandemic, implement an interprofessional student research team, and generate a meaningful dataset that is used to inform initiatives that improve student health. This report describes the protocol of the HOLISTIC Cohort Study, including survey development, recruitment strategy, and data management and analysis.

**Methods:**

An interprofessional student research team has been organized with the goal of providing continuous assessment of study design and implementation across the seven health science colleges (applied health sciences, dentistry, medicine, nursing, pharmacy, public health, and social work) at the University of Illinois Chicago in Chicago, IL. To be eligible to participate in the HOLISTIC Cohort Study, students are required to be 1) age 18 years or older; 2) enrolled full- or part-time in one or more of UIC’s seven health science colleges; and 3) enrolled in a program that prepares its graduates to enter a healthcare profession. The study protocol includes a series of three recruitment waves (Spring 2021 [April 14, 2021, to May 5, 2021; completed], Spring 2022, Spring 2023). In the first recruitment wave, eligible students were sent an invitation via electronic mail (e-mail) to complete an online survey. The online survey was based on the U.S. Centers for Disease Control and Prevention’s Behavioral Risk Factor Surveillance System 2019 survey and the 2014 World Health Organization Report of the Strategic Advisory Group of Experts Working Group Vaccine Hesitancy Scale. Electronic informed consent and study data are collected and managed using Research Electronic Data Capture (REDCap) tools. This study utilizes convenience sampling from all seven health science colleges at UIC with a target recruitment total of 2,000 participants.

**Discussion and future directions:**

A total of 555 students across all seven health science colleges (10.8% of 5,118 students who were invited; 27.6% of target sample size) enrolled in the cohort during the first recruitment wave. The pilot data establishes the feasibility of the study during the COVID-19 pandemic. Adaptations to overcome barriers to study implementation, including the use of remote, rather than in-person, study meetings, staff training, and participant recruitment are discussed. For the second and third waves of recruitment, the student research team will seek institutional review board (IRB) approval to implement additional enrollment strategies that are tailored to each health science college, such as online newsletters, virtual townhalls, flyers on bulletin boards near classrooms tailored to each health science college.

## Introduction

Prospective cohort studies examining health professionals have been crucial to the understanding of health determinants and have directly shaped healthcare interventions. For example, the British Doctors Study investigated the effects of smoking tobacco on mortality, myocardial infarction, lung cancer, and lung disease [1], the Nurses’ Health Studies established recommendations regarding women’s health [2], the Physicians’ Health Study established guidelines to prevent cardiovascular disease and cancer [3], and the Health Professionals Follow-Up Study has made numerous conclusions about the health of male dentists, pharmacists, optometrists, osteopath physicians, podiatrists, and veterinarians [4].

Cross-sectional studies of students in the health professions have similarly examined various health-related topics, including health literacy [5], use of e-cigarettes [6], empathy [7], and mental health [8,9]. In 2020, particular attention has been paid to how the coronavirus disease 2019 (COVID-19) pandemic has affected the didactic and experiential learning environment of health professional students. A National Academies of Sciences, Engineering, and Medicine workshop report in 2020 highlighted the grand challenges facing health professions education (HPE) during the COVID-19 pandemic [10]. For example, clinical training of dental and medical students temporarily relied on case presentations or remote learning, rather than experiential learning alongside practicing clinicians due to infection control precautions [11,12]. The effects of such changes in clinical training on career choices and trajectories are unknown.

Data from longitudinal cohort studies of health professional students suggest adverse consequences of the COVID-19 pandemic on mental health [13,14]. However, there is a paucity of information about changes over time across a more comprehensive set of health indicators such as physical, mental, and social health, health-related behaviors, and occupational trajectories as students are trained and enter the health professional workforce during the COVID-19 pandemic. The Health Professional Students at the University of Illinois Chicago (HOLISTIC) Cohort Study will prospectively track student participants across the seven health science colleges (applied health sciences, dentistry, medicine, nursing, pharmacy, public health, and social work) at the University of Illinois Chicago (UIC), an urban, 4-year, public university in Chicago, Illinois, USA. The HOLISTIC Cohort Study is conducted by an interdisciplinary team of students and faculty across the seven health sciences. The primary objective of the HOLISTIC Cohort Study is to characterize the health, health behaviors, and occupational trajectories of students enrolled across the seven health sciences colleges of UIC during the COVID-19 pandemic.

The purpose of this report is to describe the rationale and design of the HOLISTIC Cohort Study, including recruitment strategy, survey development, and data management. Challenges to developing a high-functioning study team during the COVID-19 pandemic and adaptations that were employed to overcome infection control-related barriers to building a research team and to recruitment of study participants are also described.

## Methods

### Overview

The HOLISTIC Cohort Study will include a total of three yearly waves of participant recruitment. The total project period will be three years and a common date will be used for close-out of study activities. Ethical approval was obtained from the Institutional Review Board of the University of Illinois Chicago with protocol #2021–0114. Written consent was obtained by participants electronically at the time of survey completion. The Interprofessional Practice and Education (IPE) program within the UIC Office of Vice Chancellor for Health Affairs (OVCHA) provides overall project oversight. A preliminary, pre-peer review version of this report is available through an online archive and distribution server [15].

The design and implementation of the HOLISTIC Cohort Study is supported by faculty mentors in three health science colleges (MK, Assistant Vice Chancellor for IPE and Associate Dean for Clinical Affairs in the College of Applied Health Sciences; JK, Associate Vice Chancellor for Population Health Sciences, Professor in the College of Medicine; and RA, Associate Dean for Academic Affairs, Professor in the School of Public Health). The faculty mentors’ initial focus was on the development of an interprofessional student research team to design and implement the HOLISTIC Cohort Study. These mentors work closely with the deans and faculty of each UIC health science college to receive continuous feedback regarding the study’s implementation and assist in developing design improvements in conjunction with the student research team.

### Development of student research team

The formation of the HOLISTIC Cohort Study’s student research team began with the recruitment of two student leaders from the College of Medicine and College of Applied Health Sciences who formed the administrative workgroup of the student research team. The administrative workgroup then began recruiting other health professional students across the seven health science colleges through an interest form that was sent via e-mail to all health professional students. The objective was to have two to three student liaisons per health science college.

The student research team is comprised of an interprofessional group representing the following UIC health science colleges: College of Pharmacy, School of Public Health, College of Nursing, College of Applied Health Sciences, College of Dentistry, College of Medicine, and Jane Addams College of Social Work. Forming an interprofessional team is crucial to the success of the project since each team member brings a unique perspective from the lens of their health profession. This fact contributes to the novelty of the study since the perspectives were synthesized during the development of all aspects of the study methodology. The student research team has developed a collaborative practice through innovation and learning during the process. As student research team members graduate, new students will be recruited in their place to continue project management.

The team is organized into five workgroups with defined responsibilities, as shown in Table 1, based on student preferences and to ensure that each workgroup included students from at least two health science colleges. The five workgroups include administrative, recruitment and retention, external communication, survey, and regulatory groups.

**Table 1:** Organization of the HOLISTIC Cohort Study research team.

Workgroup	Role
Administrative	Serve as liaisons to coordinate tasks and timelines among workgroups, facilitate communication with UIC stakeholders, meet with faculty mentors twice a month to assess progress and establish future directions, recruit new student researchers, and act as project managers
Recruitment & Retention	Create recruitment materials such as e-mail messages, flyers, study overview document, compile listservs for each eligible health science program, organize recruitment timeline for study roll-out, and develop retention strategies
External Communication	Curate the HOLISTIC Cohort Study website, collaborate with deans of each health science college to communicate study needs, and raise awareness of the project through social media and news outlets
Regulatory	Prepare and manage IRB materials, develop necessary amendments, and create future submissions
Survey	Locate existing questionnaires that can be adapted for this project, organize and update the study survey, build the online REDCap infrastructure, and manage testing and troubleshooting of survey links

### Study participant eligibility criteria

To be eligible to enroll in the HOLISTIC Cohort Study, students are required to be 1) age 18 years or older; 2) enrolled full- or part-time in one or more of UIC’s seven health science colleges; and 3) enrolled in a program that prepares its graduates to enter a healthcare profession.

### Recruitment of study participants

Enrollment in the HOLISTIC Cohort Study is designed to occur over a series of three recruitment waves (Spring 2021 [April 14, 2021 to May 5, 2021; completed], Spring 2022, Spring 2023). To ensure representation across every UIC health science college, the team is enrolling a convenience sample of students in each college that is proportional to the number of part- and full-time students in each college; the total student participant recruitment goal has been set at 2,000 participants based on available resources such as the enrollment window and funds to support reimbursement of study participants (Table 2). Recruitment procedures are designed to minimize in-person interactions between the target population of students and the student research team to reduce the risk of COVID-19 exposure, the risk of coercion or undue influence that may occur among peers, and the possibility for social desirability bias when providing responses to survey items. Thus, the study is being administered using Research Electronic Data Capture (REDCap), a secure, widely utilized web-based software platform designed to support data capture for research studies [16]. For this study, the REDCap platform includes all recruitment materials, prescreening questions, electronic informed consent (e-consent), and the HOLISTIC Cohort Study survey.

**Table 2:** Target enrollment, by UIC health science college.

University of Illinois Chicago, Health Science College	Students enrolled part- or full-time, Fall 2020, number	Eligible students, number	Target enrollment, number
College of Applied Health Sciences	2,111	462	181
College of Medicine	1,487	1,264	494
College of Dentistry	473	378	148
College of Nursing	1,504	1,398	546
College of Pharmacy	863	742	290
School of Public Health	821	396	155
Jane Addams College of Social Work	499	478	186
**Total**	7,758	5118	2,000

The University of Illinois Chicago (UIC) includes seven health sciences colleges. In the Fall 2020, there were a total of 7,758 part- and full-time students enrolled across the health sciences colleges, of which 5,118 (66%) were eligible to participate in the HOLISTIC Cohort Study (see Methods; Source Fall 2020 UIC OIR Census File). The target enrollment in the HOLISTIC Cohort Study is 2,000 health sciences students, representing about 39% of eligible students in each college.

The following steps are employed to recruit subjects:

1. *Recruitment e-mails*: Students in eligible programs receive an e-mail sent by their educational program’s listserv administrator detailing the study and eligibility requirements and providing a REDCap link inviting them to learn more about the study. The survey is active for 14 days from when the subject receives the initial recruitment e-mail. There are two reminder e-mails, one that is sent 5 days after the initial recruitment e-mail and a second, final, reminder e-mail that is sent two days before the survey is to close. Recruitment during the first wave of the study was staged one college at a time over a 3-week period to allow for the student research team to troubleshoot survey issues early in the recruitment process.
2. *Study overview*: The study overview document is the first page that participants see on the REDCap platform. This document includes the study’s purpose, procedures, potential risks and benefits, contact information for questions, and a link to guide participants to the screening questions.
3. *Screening questions*: While the initial recruitment e-mail is only sent to eligible students, two screening questions are required to confirm that participants are 18 years or older and are enrolled in at least one of the eligible degree programs based on self-report. Eligible participants are directed to an e-consent document on REDCap.
4. *E-consent*: Participants are invited to read IRB-approved text in English and to provide e-consent for the research study using the University-supported e-consent platform provided by REDCap. Due to the potential harms of asking participants to receive and send paper documents by mail during the COVID-19 pandemic and the expected scale of this project, the consent process is to be performed via e-consent. All enrolled participants receive a copy of the signed and dated consent document via e-mail.
5. *Survey completion*: Participants who offer e-consent are directed to the survey. After completing the survey, participants receive an e-mail with a $5 e-gift card as compensation for the time spent completing the survey.

### Follow-up and retention

The HOLISTIC Cohort Study is designed to recruit new participants on an annual basis for three years (2021, 2022, 2023); thus follow-up period will vary according to the date of enrollment, with those enrolled in 2021, for example, having 2 years of follow-up and those enrolled in 2023 having no additional follow-up. Follow-up participants will be invited via e-mail using their primary e-mail addresses to complete follow-up survey(s) at the same time as emails are used to enroll new participants in waves 2 and 3. In the case of no response, participants’ secondary emails and/or cell phone numbers will be used to initiate follow-up 5 to 14 days after initial email. On REDCap, participants will be asked to authenticate their identity by entering first and last name and phone number. Follow-up participants will be offered the same incentives for completing the questionnaire ($5 e-gift card) as newly enrolled participants who complete a survey.

A multi-pronged strategy will be used to promote retention of study participants throughout the three-year study period, including: 1) collecting full name, university identification number, health science college, personal e-mail address, and phone number of study participants for future follow-up 2) presenting summary results at student and faculty events, and 3) sharing information on a study website [17]. Specifically, Tableau, a data visualization software, will be used to outline selected, deidentified results from each year of the survey among all study participants; access to Tableau will be provided to all study participants once data accuracy is confirmed. This approach may motivate participants to continue filling out the survey at each subsequent year to continue learning from each year’s results.

Furthermore, input from the interprofessional student research team will be crucial to identify retention strategies, namely college-specific strategies, given their firsthand experience navigating each respective college. This strategy highlights the benefit in incorporating a multidisciplinary approach to protocol design. Input from participants in the health science colleges will additionally be collected.

### Survey development

The HOLISTIC Cohort Study survey collects participant contact information, participant demographics, and responses to questions about health and health related behaviors including vaccination (Table 3). Completion of the survey is anticipated to take about 15 to 20 minutes based on pilot testing by the student research team.

**Table 3:** HOLISTIC Cohort Study survey domains.

CATEGORY	SURVEY DOMAINS*
**General Information**	Students’ identifiers, UIC health science college enrollment, degree program enrollment
**Demographics**	Sex at birth Core section 8: Race/ethnicity, marital status, education level, rent or own residence, zip code, type of telephone number, cellphones, military service, employment status, number of children leaving in the household, annual household income
**Health status and health-related behaviors**	Core section 1: Health status Core section 2: Healthy days Core section 3: Healthcare access Core section 4: Hypertension awareness Core section 5: Cholesterol awareness Core section 6: Chronic health conditions Core section 8: Weight, height, pregnant, deaf, blind, difficulty concentrating, difficulty walking, difficulty dressing, difficulty doing errands Core section 9: Tobacco use Core section 10: Alcohol consumption Core section 11: Exercise Core section 12: Fruits & vegetable consumption Core section 13: Immunizations Optional module 1: Prediabetes, Optional Module 2: Diabetes Optional modules 5 (HPV), 6 (Flu), 7 (Shingles): Vaccinations Optional module 9: Breast & cervical cancer screening Optional module 12: Colorectal cancer screening Optional module 22: Adverse childhood experiences Optional module 25: Marijuana use Optional module 29: Sexual orientation & gender identity Vaccine hesitancy

*Core sections and Optional modules refer to the 2019 Behavioral Risk Factor Surveillance System Survey; vaccine hesitancy was assessed using the WHO SAGE Working Group Vaccine Hesitancy Scale (see Methods).

The survey is based on two validated questionnaires: 1) the U.S. Centers for Disease Control and Prevention’s (CDC) Behavioral Risk Factor Surveillance System (BRFSS) 2019 survey [18], and 2) the 2014 World Health Organization Report of the Strategic Advisory Group of Experts (WHO SAGE) Working Group Vaccine Hesitancy Scale (VHS) [19,20]. Using a deliberative process, the student research team and faculty mentors identified 12 of 14 core sections of the BRFSS for inclusion in the survey, removing “Arthritis” and “HIV/AIDS” core sections due to concerns about relevance to target population and the need for additional documentation and consent from participants given the sensitive nature of the questions, respectively. The student research team and faculty mentors also elected to include 10 of 31 optional BRFSS modules that reflect national leading health indicators as established by programs such as Healthy People 2020 or the Chicago Health Survey [21,22]. The 2014 WHO SAGE Working Group Vaccine Hesitancy Scale was used to examine vaccine hesitancy among health professional students [19,20]. Vaccine hesitancy is a barrier to controlling the spread of COVID-19, and the VHS has been used to estimate the rate of vaccine hesitancy across the globe.

The student research team made minor modifications to the instructions, wording of the questions, and response options of the BRFSS and VHS for clarity of administration via an online portal, rather than the telephone administration method used by the BRFSS. The team did not modify the original intent of questions so that the validity could be kept intact. Additional questions were added to the survey to collect contact information, the participant’s college of enrollment and degree program, and feedback about the survey. The HOLISTIC Cohort Study survey is presented in Appendix 1 (tracked changes from original BRFSS survey) and Appendix 2 (clean copy).

### Adaptations of the HOLISTIC Cohort Study to increase feasibility during COVID-19 pandemic

Infection control precautions at UIC limit opportunities for in-person meetings and collaboration. In-person recruitment via the classroom, at research fairs, or at campus events was not feasible during the first wave of recruitment. Therefore, it was necessary to develop a high-functioning study team with students and faculty responsible for the design and implementation of the IRB-approved study protocol through remote deliberations. The team used a video conferencing platform to facilitate all-team and working group meetings (Zoom Meetings, San Jose, California), e-mail for pre- and post-meeting discussions, and a cloud-based content management, collaboration, and file sharing tools (Box, Redwood City, California). To facilitate recruitment of student participants, the research team obtained IRB approval to use e-mails and a dedicated website for communication with prospective participants [17], obtain informed consent using an e-consent platform available through REDCap (Vanderbilt University), and collect questionnaire data using an electronic data capture tool, REDCap (see Data Management below).

### Data management

Study data are collected and managed using REDCap electronic data capture tools hosted at UIC [16]. REDCap surveys from each respective health science college will be exported into a Statistical Analysis System (SAS) dataset, with data labels and variable names provided for each question from the survey. Datasets will be directly imported into SAS v9.4, and additional data cleaning will be done to identify unusual responses and deal with missing data patterns. Upon completion of cleaning data, the study team will re-check all variables to make sure all responses are valid. The data will be stored with a data identifier to uniquely identify an object with a study ID.

### Data analysis

All analyses are conducted using SAS v9.4. Respondents are defined as the number of participants who complete one or more questions in the survey and click the “submit” button on the REDCap survey. The proposed target sample size of 2,000 participants was based on available resources to support enrollment and completion of follow-up visits over a three-year period. Sample size and power analysis to test specific hypotheses will be used to justify study activities beyond the three-year project period. Continuous variables will be reported using means and standard deviations, while categorical variables will be reported using counts and percentages. A one-way ANOVA hypothesis test will be performed on continuous variables to assess if the variable means between each health science group are equal or different from each other. Additionally, a chi-squared test of independence will be performed on categorical variables to assess whether there is a statistically significant relationship between groups. For categories with counts less than 5, a Fisher’s exact test will be used to assess significance between groups.

Missing data is common in cohort studies. To evaluate the pattern of missingness (e.g., missing at random (MAR), missing not at random (NMAR)), Little’s Missing Completely at Random (MCAR) Test will be performed using logistic regression with missing data pattern as outcome and a selection of covariates as predictors [23].

### Ethical considerations and dissemination

This study has sought to minimize the degree of risk associated with involvement, and procedures will be employed to minimize the risk of disclosure. All data collected will be treated with strict adherence to professional standards of confidentiality. Study records maintained in the REDCap database hosted by UIC will be password-protected and have limited access based on study role. Any unanticipated problems will be reported as per university policy.

Some of the questions are of a personal nature, and participants may feel uncomfortable by answering them. Further, although the survey is designed to collect only the minimum necessary information, subjects may consider the survey to be burdensome. Participants are free to choose which items they want to answer and which they would like to skip without affecting their relationship with the University. Resources (e.g., mental and physical health referrals) are also provided for those individuals who request support.

Full results for this project will be disseminated to internal stakeholders (e.g., students, college administrators, and faculty) through websites, newsletters, and virtual townhalls. Dissemination to external stakeholders will be through peer-reviewed publications and presentations at regional and national meetings.

## Results

This study began recruitment on April 14, 2021 and is ongoing, with enrollment anticipated to continue through April 2023. Of the 5,118 eligible students across all seven health sciences colleges who were invited to participate in the first wave of recruitment, 555 students (10.8%) enrolled. These participants matriculated at all seven UIC health science colleges (Table 4). The average age of currently enrolled student participants is 28.1 years, 438 (79.1%) identify as female, and 138 (24.9%) identify as an underrepresented minority, defined as Hispanic, Black/African American, American Indian/Alaskan Native, and Multi-race (Table 5).

**Table 4:** Degree programs among HOLISTIC Cohort Study participants enrolled as of May 5, 2021 (after first of three recruitment waves).

Health Science College/Degree Program	N (%)
**Applied Health Sciences**	
BS: Health Information Management	10 (10.8%)
BS: Health Information Management (Online)	2 (2.1%)
BS: Nutrition-Coordinated Program	2 (2.1%)
MS: Nutrition	11 (11.7%)
DPT: Physical Therapy	42 (45.2%)
MS: Occupational Therapy	26 (28.0%)
**Total**	**93 (100.0%)**
**Dentistry**	
DMD: Dental Medicine	35 (7.8%)
DMD-AS: Dental Medicine-Advanced Standing	10 (22.2%)
**Total**	**45 (100.0%)**
**Medicine**	
MD: Medicine—Chicago	75 (57.2%)
MD: Medicine—Peoria	24 (18.3%)
MD: Medicine—Rockford	32 (24.4%)
MD: Medicine—Urbana	0 (0.0%)
**Total**	**131 (100.0%)**
**Nursing**	
BSN: Registered Nurse to Bachelor of Nursing—Chi	2 (2.0%)
BSN: Nursing—Chicago	16 (15.8%)
BSN: Nursing—Springfield	0 (0.0%)
BSN: Nursing—Urbana	9 (8.9%)
MS: Nursing—Chicago	39 (38.6%)
MS: Nursing—Urbana	2 (2.0%)
DNP: Nursing Practice—Chicago	25 (24.8%)
DNP: Nursing Practice—Peoria	3 (3.0%)
DNP: Nursing Practice—Quad Cities	0 (0.0%)
DNP: Nursing Practice—Springfield	2 (2.0%)
DNP: Nursing Practice—Urbana	0 (0.0%)
DNP: Nursing Practice—Rockford	3 (3.0%)
**Total**	**101 (100.0%)**
**Pharmacy**	
PharmD: Pharmacy—Chicago	62 (72.9%)
PharmD: Pharmacy—Rockford	23 (27.1%)
**Total**	**85 (100.0%)**
**Public Health**	
MHA: Healthcare Administration	6 (12.8%)
MPH: Community Health Sciences	16 (34.0%)
MPH: Community Health Sciences (Online)	2 (4.3%)
MPH: Public Health Informatics	0 (0.0%)
MPH: Health Policy & Administration	8 (17.0%)
MPH: Health Policy & Administration (Online)	0 (0.0%)
MPH: Environmental & Occupational Health Sciences	2 (4.3%)
MPH: Biostatistics	1 (2.1%)
MPH: Epidemiology	12 (25.5%)
**Total**	**47 (100.0%)**
**Social Work**	
MSW: Social Work—UIC	49 (100.0%)
**Total**	**49 (100.0%)**

The number of participants contributing to information in the Table may be less than N = 555, if a participant did not respond to a specific question.

**Table 5:** Baseline characteristics of participants enrolled as of May 5, 2021 (after first of three recruitment waves).

Characteristic	Applied Health Sciences	Dentistry	Medicine	Nursing	Pharmacy	Public Health	Social Work	Total
**Age (Years)**: Mean ± SD	26.5 ± 3.4	29.9 ± 5.6	27.4 ± 2.3	29.6 ± 7.4	26.9 ± 3.2	28.0 ± 5.3	30.3 ± 6.6	28.1 ± 5.0
**Gender**: N (%)								
Male	17 (18.3%)	32 (72.7%)	45 (33.8%)	7 (6.9%)	26 (30.6%)	3 (6.3%)	2 (4.0%)	111 (20.0%)
Female	76 (81.7%)	11 (25.0%)	86 (64.7%)	93 (92.1%)	58 (68.2%)	45 (93.7%)	48 (96.0%)	438 (79.1%)
Other Response	0 (0.0%)	1 (2.3%)	2 (1.5%)	1 (1.0%)	1 (1.2%)	0 (0.0%)	0 (0.0%)	5 (0.9%)
Underrepresented Minority*: N(%)								
Yes	16 (17.2%)	10 (22.2%)	33 (24.8%)	20 (80.2%)	23 (27.1%)	16 (33.3%)	20 (40.0%)	138 (24.9%)
No	77 (82.8%)	35 (77.8%)	100 (75.2%)	81 (19.8%)	62 (72.9%)	32 (66.7%)	30 (60.0%)	417 (75.1%)

*Underrepresented minority defined as Hispanic, Black/African American, American Indian/Alaskan Native, or Multi-race; the number of participants contributing to information in the Table may be less than N = 555, if a participant did not respond to a specific question.

## Discussion

The HOLISTIC Cohort Study is designed to offer information about the physical, mental, and social health and occupational trajectory of students trained during the pandemic at an urban public university as they enter the health professional workforce over the next few years. Information about the participants enrolled after the first recruitment wave should be considered preliminary, as we intend to describe the baseline characteristics of the full study cohort in a separate publication after all three waves of recruitment have been completed. Just over one-quarter (27.6%) of the 2,000-participant enrollment target has been enrolled after the first of three waves of recruitment, supporting the feasibility of the study during the COVID-19 pandemic.

There are several unique aspects of the HOLISTIC Cohort Study design. First, the study is being designed by an interprofessional student research team, supporting their professional and educational development through interprofessional collaboration. Second, the design and implementation of the study including recruitment, enrollment, and completion of the survey is being conducted entirely online through virtual study team meetings, electronic approaches to participant recruitment, and use of e-consent due to infection control precautions during the COVID-19 pandemic. In addition, the HOLISTIC Cohort Study uses questions based on the BRFSS and VHS, which allows all data collected throughout the course of the study to be compared with results from other studies and, in the case of the BRFSS, benchmarked with state and national trends in the U.S. Lastly, this study includes study participants across seven UIC health science colleges, which provides a unique opportunity to compare the health, health-related behaviors, and occupational trajectories across a wide interprofessional spectrum of health professions trained during the COVID-19 pandemic.

### Study limitations

During the first wave of recruitment, the primary challenge that was observed was a low response rate (about 11%). The team relied exclusively on e-mails to students and were unable to use in-person events or campaigns to raise awareness of the study due to infection control precautions. For the second and third waves of recruitment, the student research team will seek IRB approval to implement online newsletters, social media, class announcements, and virtual townhalls to improve visibility of the study. These approaches may need to be tailored to each health science college. Also, the study participants are from a single, minority-serving public university; it is not known if findings from the HOLISTIC Cohort Study would apply to health sciences students elsewhere. Multi-center studies that employ the protocol outlined in this report across multiple public and private universities in the U.S. and elsewhere could be used to address the potential for limited generalizability from the HOLISTIC Cohort Study.

### Current status and timeline

The HOLISTIC Cohort Study began enrolling its first round of participants in Spring 2021, with 553 student participants currently enrolled. The second and third rounds of recruitment, which will include additional strategies to reach the target student population, are expected to launch in Spring 2022 and Spring 2023. The total project period will be three years and a common date will be used for close-out of study activities. Thus, follow-up period will vary according to the date of enrollment (enrolled in wave 1: 2 years of follow-up; enrolled in wave 2: 1 year of follow-up; enrolled in wave 3: no additional follow-up). Sample size and power analysis to test specific hypotheses will be used to justify study activities and funding beyond the three-year project period.

## Conclusion

The HOLISTIC Cohort Study has established a platform for assessing the health of health professional students educated during a unique time in history—the COVID-19 pandemic. The longitudinal design will allow evaluation of changes across a comprehensive set of health indicators (physical, mental, and social health and health-related behaviors) and occupational trajectories as students trained during the COVID-19 pandemic enter the health professional workforce. Adaptations were employed to overcome infection control-related barriers to building a research team and to recruitment of study participants, including the use of video conferencing, e-consent, and a cloud-based content management system.

## Supporting information

S1 Appendix (DOCX)
